# Assessment of Depressive and Anxiety Symptoms and Health-Related Quality of Life in Rosacea Patients: A Case-Control Study

**DOI:** 10.1155/drp/5532532

**Published:** 2024-12-07

**Authors:** Khaled Seetan, Mohammad Gablan, Mohammad Alnaimi, Daleen Alhazaimeh, Mohammad Bani Younes, Ahmad Alnaimi, Almutazballlah Qablan

**Affiliations:** ^1^Department of Clinical Sciences, Faculty of Medicine, Yarmouk University, Irbid, Jordan; ^2^Department of Clinical Sciences, Faculty of Medicine, Jordan University of Science and Technology, Irbid, Jordan

**Keywords:** anxiety, depression, quality of life, rosacea, well-being

## Abstract

**Background:** Rosacea, a chronic facial dermatosis, poses a substantial global prevalence burden. Its impact extends beyond physical symptoms, affecting patient quality of life, self-esteem, and psychosocial functioning. This study aims to assess the health-related quality of life and emotional well-being in Jordanian rosacea patients in comparison to healthy controls.

**Methods:** A case-control study, matching for age and sex, was conducted among rosacea patients attending the outpatient department at a governmental secondary hospital in the north of Jordan between September 2022 and November 2023. Adult patients with a confirmed rosacea diagnosis for a duration of ≥ 3 months were paired with healthy controls. Health-related quality of life, anxiety, and depression were evaluated using the Dermatology Life Quality Index (DLQI) and the Hospital Anxiety and Depression Scale (HADS), respectively. Disease severity was assessed using the Clinician's Erythema Assessment (CEA) grading system.

**Results:** The study included 198 rosacea patients and 198 healthy controls. Rosacea patients exhibited significantly higher DLQI scores (mean 11.32 ± 5.4) compared to controls (mean 4.28 ± 2.3), indicating diminished quality of life. Subscale analysis revealed prominent differences in symptoms/feelings and daily activities. Additionally, rosacea patients reported elevated scores on the HADS anxiety (mean 9.38 ± 3.2) and depression (mean 8.19 ± 4.3) subscales in contrast to controls (mean 3.88 ± 2.18 and mean 3.41 ± 1.87, respectively). More than half (57%) of rosacea patients experienced moderate or severe anxiety, and nearly a third (31%) reported moderate or severe depression. Regression analyses revealed that higher disease severity significantly predicted worse quality of life (*β* = 0.384), anxiety (*β* = 0.352), and depression (*β* = 0.312).

**Conclusion:** The study demonstrated that rosacea patients in Jordan experience significantly diminished quality of life and higher rates of anxiety and depression compared to healthy controls. Disease severity and multiple facial lesions emerged as strong predictors of poor psychological outcomes, while female gender increased vulnerability and longer disease duration showed a protective effect. These findings emphasize the need for routine psychological screening and a holistic treatment approach, particularly for newly diagnosed patients with severe disease manifestations.

## 1. Introduction

Rosacea is a chronic facial dermatosis with an estimated worldwide prevalence of 5%, ranging from 2% to over 10% in different populations [1, 2]. It is estimated to impact over 16 million Americans [3] and approximately 10% of the European population [4]. Prominent symptoms include central facial erythema, inflammatory papules and pustules, telangiectasia, burning or stinging sensations, and ocular manifestations [5]. More advanced disease can lead to phymatous changes, such as skin thickening, irregular surface nodularities, and rhinophyma [6]. Typically, rosacea first presents between the ages of 30–60 years [1]. Various potential pathophysiologic mechanisms have been proposed, including neurovascular dysregulation, immune-mediated inflammation, microbial organisms, and genetics [7, 8]. However, the etiology remains incompletely understood.

While not life-threatening, rosacea can profoundly impact patient quality of life and psychosocial functioning [9–11]. Facial redness, lesions, and disfigurement often damage self-image and self-esteem [10]. Ocular discomfort can further reduce well-being [12]. Dermatology-specific quality of life has been shown to be affected in patients with rosacea across different studies [13]. Psychiatric comorbidity also frequently accompanies rosacea. According to a meta-analysis of nine studies involving more than 100 million patients with rosacea, the condition was significantly associated with depression and anxiety, both as comorbidities and potential outcomes. This implies that patients with rosacea are more likely to have or develop depression and anxiety than those without rosacea [14]. Therefore, clinicians must consider the psychological impact of rosacea and provide appropriate support and treatment for their patients.

While rosacea's psychosocial impact has been well-documented in Western and Asian populations, there is a notable gap in understanding its effects within Middle Eastern communities. Cultural factors, social norms, and beauty standards vary significantly across regions and can substantially influence how visible skin conditions affect individuals' psychological well-being and quality of life. In Jordan and similar Middle Eastern societies, where social interactions are heavily influenced by cultural and religious practices, facial appearance holds particular significance in both personal and professional contexts. Additionally, environmental factors specific to the region, such as intense sun exposure and arid climate, may affect both the presentation and management of rosacea, potentially impacting patients' psychological burden differently than in other geographic regions.

Despite rosacea's growing public health impact and the unique sociocultural context of the Middle East, research examining quality of life and psychosocial health in this region remains scarce. Previous studies have primarily focused on Western and Asian populations, leaving uncertainty about whether their findings can be generalized to Middle Eastern communities. This study aimed to address this knowledge gap by assessing health-related quality of life and emotional well-being in Jordanian rosacea patients compared to healthy controls. By examining these outcomes in a Jordanian context, our research provides valuable insights into how cultural, environmental, and healthcare system differences might influence the psychosocial burden of rosacea. Additionally, this study sought to analyze variations in quality of life, anxiety, and depression based on sociodemographic factors and rosacea disease characteristics, including severity and morphology, to inform culturally appropriate interventions and support strategies for this population.

## 2. Materials and Methods

### 2.1. Participants

We conducted a case-control study of 198 rosacea patients presenting to the outpatient department at a government secondary hospital in the North of Jordan between September 2022 and November 2023. Inclusion criteria for cases were age 18 years and older, a clinical diagnosis of rosacea by a board-certified dermatologist, and disease duration of ≥ 3 months. Exclusion criteria included comorbid uncontrolled medical conditions (e.g., cancers and congestive heart failure), active psychiatric illness, or the use of systemic retinoid/immunosuppressant in the past month. Rosacea diagnosis was established by board-certified dermatologists. Diagnosis required either one diagnostic phenotype (fixed centrofacial erythema in a characteristic pattern or phymatous changes) or two major phenotypes (transient centrofacial erythema/flushing, inflammatory papules/pustules, telangiectasia, or ocular manifestations). Patients were classified into four subtypes: erythemato-telangiectatic, papulopustular, phymatous, or ocular rosacea, with the possibility of multiple subtypes coexisting.

The control group consisted of 198 individuals recruited from the same hospital's general outpatient clinics during routine check-ups and from hospital staff during the same time period. Controls were individually matched to cases by age (±2 years) and gender. Inclusion criteria for controls included age ≥ 18 years and absence of any chronic skin conditions. Control subjects were specifically examined by a dermatologist to confirm the absence of rosacea or other facial dermatoses. Exclusion criteria for controls matched those of cases.

### 2.2. Ethical Clearance

A written informed consent was obtained from each study participants after explaining the study purpose. Participation was totally voluntary, and the participants had the right to withdraw from the study at any time without any consequences on the health care delivery, and the data were kept confidential and used for research purposes only. The study was approved from the Yarmouk University Institutional Board Review (BD/119/12/5974).

### 2.3. Research Instruments

Data were collected using a structured, author-designed questionnaire filled out during an interview between each participant and the investigating physician. The questionnaire comprised three sections: sociodemographic characteristics, disease-related information, and the level of quality of life, anxiety, and depression. Quality of life was assessed using the Dermatology Life Quality Index (DLQI) scale, consisting of 10 items that evaluate the impact of dermatological disease on health-related quality of life across six domains: symptoms and feelings (items 1-2), daily activities (items 3–4), leisure (items 5–6), work/school (item 7), personal relationships (items 8-9), and treatment (item 10). Each question was scored on a Likert scale from 0 to 3, resulting in a total score ranging from 0 to 30. Classification was as follows: grade 1 (0-1) indicated no impact on patients' life, grade 2 (2–5) indicating low impact, grade 3 (6–10) indicating moderate impact, grade 4 (11–20) indicating a very large impact, and grade 5 (21–30) indicating an extremely large impact on patients' life. The DLQI has demonstrated excellent psychometric properties with high internal consistency (Cronbach's *α* = 0.83–0.93) [15]. Anxiety and depression levels were assessed using the Hospital Anxiety and Depression Scale (HADS) scale, comprising 14 items, anxiety (HADS-A, 7 items), and depression (HADS-D, 7 items). Each item is scored from 0 to 3, with subscale scores ranging from 0 to 21. Classification was as follows: low (0–7), moderate (8–10), or high (11 or more) for each of the anxiety and depression scales. The scale has shown robust psychometric properties (Cronbach's *α* = 0.80–0.93 for both subscales) [16]. Rosacea severity was clinically assessed using the Clinician Erythema Assessment (CEA) scale, and it is a validated tool for evaluating rosacea severity. It uses standardized photographic criteria to grade facial erythema classifying disease severity into five grades (clear, almost clear, mild, moderate, or severe) [17].

### 2.4. Data Analysis

Collected data were entered and analyzed using the Statistical Package for Social Sciences (SPSS) Version 26.0, following revision and cleaning in Microsoft Excel. Descriptive statistics were applied to all variables, with results reported as frequencies and percentages for categorical variables, and means and standard deviations (SDs) for continuous variables. Differences between categorical variables were compared using Chi-square tests, while independent samples *t*-test and one-way ANOVA were used for continuous variables to analyze the relationship between disease characteristics and outcome measures (DLQI, HADS anxiety, and HADS depression). For investigating associations between disease severity (CEA scores) and psychological outcomes, we used multiple linear regression models. A *p* value of ≤ 0.05 was considered statistically significant.

Dependent study variables included the DLQI, anxiety, and depression levels measured by the HADS. Independent variables included demographic characteristics (age, gender, marital status, educational level, monthly income, and smoking status), rosacea disease state, subtype, duration, and severity.

## 3. Results

### 3.1. Participant Characteristics

The study sample comprised 198 rosacea patients and 198 healthy controls. No significant differences were found between groups in mean age (approximately 37 years) or gender distribution (75% female). Nearly half of both groups were married. The majority had a college education, with no differences between groups. A significantly higher proportion of controls had monthly incomes exceeding 250 JDs compared to rosacea patients (*p*=0.01). Smoking rates were significantly lower in the control group (54% nonsmokers) compared to the rosacea group (36% nonsmokers, *p* < 0.001), as shown in Table 1.

### 3.2. DLQI Scores

**Figure 1:**
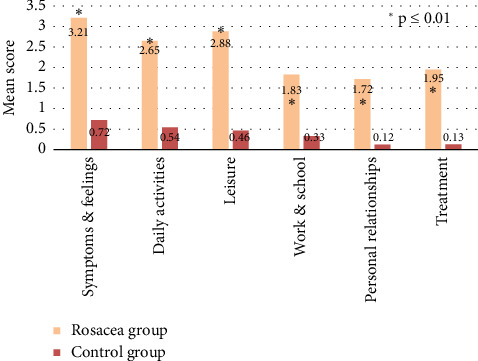
Difference between the rosacea and control groups regarding scores in the DLQI subscales.

Overall DLQI scores were significantly higher in rosacea patients (mean 11.32, SD 5.4) compared to controls (mean 4.28, SD 2.3, *p* < 0.001), indicating a poorer disease-specific quality of life. The distribution of DLQI grades also differed significantly between groups (*p* < 0.001), with the majority of controls (85%) classified as Grade 1 (“a small effect on the patient's life”) versus only 11% of rosacea patients. In contrast, over 60% of rosacea patients had Grade 3–5 DLQI scores, representing a very large, extremely large, or unbearably large effect of the disease on the quality of life. Rosacea patients demonstrated substantially reduced quality of life across all DLQI subscales compared to controls (Figure 1). The domains with the most significant differences were symptoms/feelings and the performance of daily activities.

### 3.3. HADS Scores

Mean anxiety subscale scores on the HADS were significantly higher in the rosacea cohort (mean 9.38, SD 3.2) versus controls (mean 3.88, SD 2.18, *p* < 0.001). More than half (57%) of rosacea patients had moderate or severe anxiety levels compared to 28% of controls (*p* < 0.001). Similarly, mean depression subscale scores were significantly elevated in the rosacea group (8.19, SD 4.3) relative to controls (3.41, SD 1.87, *p* < 0.001), with 31% categorized as having moderate or severe depression compared to 13% of controls (*p* < 0.001), as shown in Table 2.

### 3.4. Correlation of DLQI, Anxiety, and Depression in Rosacea Patients

**Figure 2:**
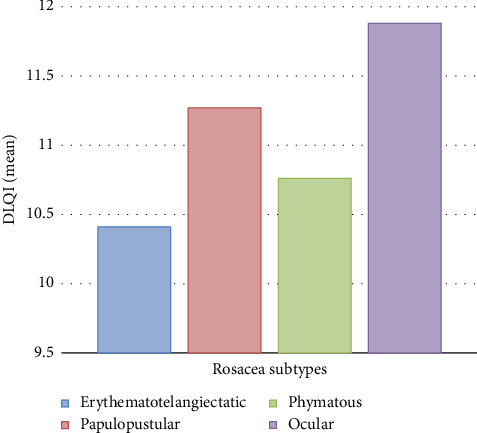
Mean DLQI scores between the different rosacea subtypes.

Concerning disease duration, patients with > 3 years of duration had significantly lower anxiety scores (mean 7.23, *p*=0.007) than those with a shorter disease duration. Patients with ≥ 4 facial lesions (123/198, 62.1%) exhibited lower mean DLQI scores (9.71 vs. 11.84, *p*=0.001) and anxiety scores (8.13 vs. 10.72, *p*=0.001) than those with < 4 lesions (75/198, 37.9%). Among rosacea subtypes, papulopustular patients had the highest DLQI scores (mean 11.27), while phymatous patients had the highest anxiety and depression scores (means 9.74 and 8.85, respectively). With increasing disease severity on the CEA scale, DLQI and anxiety scores decreased. Patients with “clear” rosacea (31/198, 15.7%) had the lowest mean DLQI (7.64) and anxiety scores (6.81) (*p*=0.001 for both compared to other severity grades), as shown in Table 3 and Figure 2.

### 3.5. Quality of Life, Depression, and Anxiety and Association With Disease Characteristics

Multiple linear regression analysis revealed significant associations between disease characteristics and psychological outcomes (Table 4). Higher CEA severity scores were independently associated with worse DLQI scores (*β* = 0.384, *p* < 0.001), increased anxiety (*β* = 0.352, *p* < 0.001), and depression (*β* = 0.312, *p* < 0.001). The presence of ≥ 4 facial lesions was also significantly associated with poorer outcomes across all measures (DLQI: *β* = 0.329, *p* < 0.001; anxiety: *β* = 0.295, *p* < 0.001; depression: *β* = 0.275, *p* < 0.001). Interestingly, longer disease duration showed a small protective effect, with negative associations across all outcomes. Female gender was associated with worse scores on all measures, while age showed no significant associations. These relationships remained significant after adjusting for education level and monthly income.

## 4. Discussion

The present study provides compelling evidence that rosacea has a profound adverse impact on disease-specific quality of life. Rosacea patients demonstrated significantly poorer quality of life on the DLQI compared to healthy controls. The mean total DLQI score of 11.32 and the distribution of DLQI grades suggest extremely large impairments in multiple domains of daily living for the majority of rosacea patients. These findings align with previous global studies, including those conducted in China (mean DLQI 11.6 and 12.6) [18, 19] and the UK (mean DLQI 17.3) [20]. Thus, our results corroborate that rosacea represents a distressing condition with pervasive negative impacts across various countries and cultures. However, conflicting findings from other studies reporting lower DLQI scores in Ireland (mean DLQI 5.2) [21], Germany (mean DLQI 4.05) [22], China (mean DLQI 7.59) [23], this may be attributed to population differences and variability of disease severity between the studies. Moreover, reports from other countries [13] with average DLQI scores between 2.5 and 5.6 may stem from differences in the methodological assessment of quality of life and rosacea, as well as potential selection bias.

Our study offers robust evidence that rosacea negatively influences quality of life and psychosocial well-being. Rosacea patients had significantly higher mean DLQI scores across all domains compared to controls, indicating substantial effects on symptoms, daily activities, leisure, relationships, and more [24]. The domains with the most significant differences were symptoms/feelings and performance of daily activities, aligning with prior research demonstrating that facial redness and flushing provoke distress across different cultures and geographic regions [5, 21, 25].

Over half of Jordanian rosacea patients in our sample had moderate or severe anxiety based on HADS scores. This extends previous observations of anxiety prevalence of 24.7% in rosacea populations [26]. Plausible explanations include social anxiety related to facial disfigurement, anxiety triggered by flushing episodes, stigma related to appearance, or underlying inflammatory pathways influencing mental health [27]. Similarly, the depression rate among our rosacea cohort (31% moderate/severe) was significantly elevated compared to controls, consistent with previous evidence [10].

We also identified important clinical characteristics associated with poorer quality of life, anxiety, and depression among rosacea patients. Those with more facial lesions, papulopustular subtype, and mild-moderate CEA severity tended to have worse scores across all metrics. This is consistent with previous observations that severe symptomatic rosacea imposes a significant psychological burden [28]. It is worth noting that no significant association found between the disease duration and increased anxiety scores. Our study contributes uniquely to this evidence base through the inclusion of a matched control group for contextualized comparisons.

Our findings have several important implications for clinical practice. The high prevalence of anxiety (57%) and depression (31%) among rosacea patients suggests the need for routine psychological screening in dermatology clinics using brief tools like HADS. A multimodal treatment approach combining physical symptom management with psychological support is recommended, particularly for patients with visible facial lesions and higher disease severity. Early referral to mental health professionals should be considered for patients showing significant psychological distress. Patient education should include both skincare management and coping strategies for psychosocial challenges. Special attention should be paid to newly diagnosed patients, who may be more vulnerable to psychological distress compared to those with longer disease duration. These interventions should be tailored to the Jordanian cultural context, where facial appearance significantly impacts social interaction.

While promising, these findings should be interpreted within the limitations inherent to case-control design. First, the hospital-based recruitment of cases and controls may limit generalizability to the broader population, particularly those not seeking medical care. Second, despite matching for age and gender, unmeasured confounding factors could influence the observed associations such as lifestyle factors and stress levels. Finally, longitudinal studies are needed to determine the temporal relationship between rosacea and psychological distress.

## 5. Conclusion

The study demonstrated that rosacea patients in Jordan experience significantly diminished quality of life and higher rates of anxiety and depression compared to healthy controls. Disease severity and multiple facial lesions emerged as strong predictors of poor psychological outcomes, while female gender increased vulnerability and longer disease duration showed a protective effect. These findings emphasize the need for routine psychological screening and a holistic treatment approach, particularly for newly diagnosed patients with severe disease manifestations.

## Figures and Tables

**Table 1:** Sociodemographic characteristics of study participants.

		Study group				*p*(*X*^2^/*t*)
		Rosacea (*n* = 198)		Control (*n* = 198)		
		Mean (SD)	*N* (%)	Mean (SD)	*N* (%)	
Age in years		37.3 (13.4)		37.5 (13)		0.59 (0.49)

Gender	Female		155 (78.3%)		147 (74.2%)	0.66 (0.12)
	Male		43 (21.7%)		51 (25.8%)	

Marital status	Married		93 (47.0%)		88 (44.5%)	0.7 (0.67)
	Single		78 (39.4%)		87 (43.9%)	
	Widowed		27 (13.6%)		23 (11.6%)	

Educational level	Secondary or below		37 (18.7%)		29 (14.6%)	0.62 (0.47)
	College diploma		143 (72.2%)		150 (75.8%)	
	Higher education		18 (9.1%)		19 (9.6%)	

Monthly income	Less than 250 JDs		43 (21.7%)		70 (35.3%)	0.01^∗^ (8.5)
	Between 250 and 750 JDs		137 (69.2%)		113 (57.1%)	
	More than 750 JDs		18 (9.1%)		15 (7.6%)	

Smoking status	Smoker		72 (36.4%)		91 (46.0%)	0.023^∗^ (5.38)
	Nonsmoker		126 (63.6%)		107 (54.0%)	

^∗^Statistical significance at *p* ≤ 0.05.

**Table 2:** Difference in DLQI, HADS depression, and anxiety scores between the rosacea and control groups.

		Study group				*p*(*X*^2^/*t*)
		Rosacea (*n* = 198)		Control (*n* = 198)		
		Mean (SD)	*N* (%)	Mean (SD)	*N* (%)	
Overall DLQI		11.32 (5.4)		4.28 (2.3)		< 0.001^∗^ (14.24)

DLQI grade	Grade 1		22 (11.1%)		85 (42.9)	< 0.001^∗^ (16.1)
	Grade 2		38 (19.2%)		52 (26.3%)	
	Grade 3		76 (38.4%)		49 (24.7%)	
	Grade 4		44 (22.2%)		12 (6.1%)	
	Grade 5		19 (9.6%)		0 (0%)	

Overall HADS-A		9.38 (3.2)		3.88 (2.18)		< 0.001^∗^ (9.37)

Anxiety level	Low (0–7)		83 (41.9%)		142 (71.7%)	< 0.001^∗^ (8.82)
	Moderate (8–10)		85 (42.9%)		47 (23.7%)	
	High (11 or more)		30 (15.2%)		9 (4.6%)	

Overall HADS-D		8.19 (4.3)		3.41 (1.87)		< 0.001^∗^ (6.94)

Depression level	Low (0–7)		138 (69.7%)		171 (86.4%)	< 0.001^∗^ (7.66)
	Moderate (8–10)		38 (19.2%)		19 (9.6%)	
	High (11 or more)		23 (11.6%)		8 (4.0%)	

^∗^Statistical significance at *p* ≤ 0.05.

**Table 3:** Difference in DLQI, HADS depression, and anxiety scores according to disease characteristics and severity among rosacea patients (*n* = 198).

		*N* (%)	DLQI		HADS-A		HADS-D	
			Mean (SD)	*p*	Mean (SD)	*p*	Mean (SD)	*p*
Disease duration	< 1 years	95 (48.0)	12.11 (3.95)	< 0.001^∗^	10.13 (2.97)	0.007^∗^	10.67 (3.24)	0.01^∗^
	1–3 years	58 (29.3%)	10.73 (3.51)		8.83 (2.71)		9.1 (3.14)	
	> 3 years	45 (22.7%)	8.82 (3.32)		7.23 (2.61)		8.15 (2.82)	

Rosacea signs	< 4	75 (37.9%)	11.84 (3.7)	< 0.001^∗^	10.72 (3.6)	< 0.001^∗^	10.51 (3.2)	< 0.001^∗^
	≥ 4	123 (62.1%)	9.71 (3.44)		8.13 (2.9)		7.63 (2.9)	

Rosacea subtype	Erythematotelangiectatic	103 (52.0%)	10.41 (3.82)	0.16	7.34 (2.7)	0.006^∗^	7.22 (2.31)	0.003^∗^
	Papulopustular	67 (33.8%)	11.27 (5.1)		11.46 (4.1)		10.76 (3.54)	
	Phymatous	13 (6.6%)	10.76 (4.0)		9.74 (2.5)		8.85 (3.1)	
	Ocular	15 (7.6%)	11.88 (4.52)		10.78 (2.8)		9.1 (2.9)	

CEA severity	Clear	31 (15.7%)	7.64 (2.81)	< 0.001^∗^	6.81 (2.91)	< 0.001^∗^	7.1 (2.65)	< 0.001^∗^
	Almost clear	58 (29.3%)	9.45 (3.43)		8.26 (2.77)		7.73 (2.82)	
	Mild	77 (38.9%)	11.33 (3.7)		10.63 (3.2)		9.9 (3.13)	
	Moderate	24 (12.1%)	10.92 (3.61)		9.73 (2.87)		8.82 (2.9)	
	Severe	8 (4.0%)	10.12 (3.8)		9.5 (3.0)		8.65 (2.85)	

^∗^Statistical significance at *p* ≤ 0.05.

**Table 4:** Multiple linear regression analysis of associated factors with poorer quality of life among rosacea patients (*n* = 198).

Dependent variable	Independent variables	Adjusted *β*	95% CI	*p* value
DLQI score	CEA severity score	0.384	0.245 to 0.523	< 0.001^∗^
	Number of facial lesions (≥ 4)	0.329	0.198 to 0.460	< 0.001^∗^
	Disease duration	−0.156	−0.287 to −0.025	0.019^∗^
	Age	−0.089	−0.220 to 0.042	0.182
	Female gender	0.143	0.012 to 0.274	0.033^∗^

HADS anxiety	CEA severity score	0.352	0.213 to 0.491	< 0.001^∗^
	Number of facial lesions (≥ 4)	0.295	0.164 to 0.426	< 0.001^∗^
	Disease duration	−0.178	−0.309 to −0.047	0.008^∗^
	Age	−0.102	−0.233 to 0.029	0.127
	Female gender	0.168	0.037 to 0.299	0.012^∗^

HADS depression	CEA severity score	0.312	0.173 to 0.451	< 0.001^∗^
	Number of facial lesions (≥ 4)	0.275	0.144 to 0.406	< 0.001^∗^
	Disease duration	−0.145	−0.276 to −0.014	0.031^∗^
	Age	−0.094	−0.225 to 0.037	0.159
	Female gender	0.159	0.028 to 0.290	0.018^∗^

*Note:* Statistical significance at *p* ≤ 0.05. *β* = standardized regression coefficient. The asterisk (^∗^) denotes variables with statistically significant associations, as indicated by a *p* value less than or equal to 0.05. This threshold was used to highlight factors that have a meaningful impact on the dependent variables (DLQI score, HADS anxiety, and HADS depression) in the context of this study.

Abbreviation: CI = confidence interval.

## Data Availability

The data that support the findings of this study are available from the corresponding author upon reasonable request.
